# Novel Pyrazolo[3,4-b] Pyridine Derivative (HLQ2g) Attenuates Hypoxic Pulmonary Hypertension via Restoring cGKI Expression and BMP Signaling Pathway

**DOI:** 10.3389/fphar.2021.691405

**Published:** 2021-10-01

**Authors:** Lijun Li, Minghui Yin, Liqing Hu, Xiaoting Tian, Xiangrong He, Congke Zhao, Ying Li, Qianbin Li, Xiaohui Li

**Affiliations:** ^1^ Department of Pharmacology, Xiangya School of Pharmaceutical Sciences, Central South University, Changsha, China; ^2^ Department of Medicinal Chemistry, Xiangya School of Pharmaceutical Sciences, Central South University, Changsha, China; ^3^ Department of Pharmacy, Henan Provincial People’s Hospital, People’s Hospital of Zhengzhou University, Zhengzhou, China; ^4^ Department of Health Management, The Third Xiangya Hospital of Central South University, Changsha, China; ^5^ Hunan Key Laboratory for Bioanalysis of Complex Matrix Samples, Changsha, China

**Keywords:** pulmonary hypertension, hypoxia, cGKI, BMP signaling pathway, pulmonary artery smooth muscle cells

## Abstract

Pulmonary hypertension (PH) is an extremely serious cardiopulmonary disease, finally leading to progressive right ventricular failure and death. Our previous studies have nominated HLQ2g, a pyrazolo[3,4-b] pyridine derivative stimulating soluble guanylate cyclase (sGC), as a new candidate for the treatment of PH, but the specific mechanism is still not clear. The PH model induced by hypoxia was established in rats. Right ventricular systolic pressure (RVSP) was assessed by jugular vein catheterization. RV weight was the index to evaluate RV hypertrophy. The protein levels of cGMP-dependent protein kinase type I (cGKI), bone morphogenetic protein receptor 2 (BMPR2), phosphorylated Smad1/5/8 (p-Smad1/5/8), and inhibitor of differention 1 (Id1) in pulmonary artery and human pulmonary artery smooth muscle cells (HPASMCs) were determined by western blotting. Cell proliferation and migration were evaluated. In the whole experiment, the first clinically available sGC stimulator Riociguat was used as the reference. In hypoxic PH rat model, elevated RVSP and RV hypertrophy were significantly reduced by HLQ2g treatment. Both Riociguat and HLQ2g attenuated vascular remodeling accompanied with up-regulated cGKI expression and BMP signaling pathway, which was characterized by elevated expression of BMPR2, p-Smad1/5/8, and Id1 in HPH rats. In addition, HLQ2g inhibited proliferation and migration of HPASMCs induced by hypoxia and platelet-derived growth factor (PDGF), restored BMPR2 signaling, which was recalled by Rp-8-Br-PET-cGMPS, the inhibitor of cGKI. In summary, the novel pyrazolo[3,4-b] pyridine derivative HLQ2g can alleviate HPH progression by up-regulating cGKI protein and BMP signaling pathway.

## Introduction

Pulmonary hypertension (PH) refers to the resting mean pulmonary arterial pressure ≥20 mmHg as evaluated by right heart catheterization, which has always been an important clinical challenge due to its high mortality (Simonneau et al., 2019). PH patients have a median survival time of 5–7 years, which is featured by pulmonary vascular system remodeling, resulting in decreased pulmonary arterial compliance and elevated pulmonary vascular resistance (Thenappan et al., 2019). Continuous elevation of pulmonary artery pressure increases right ventricular (RV) afterload, which may consequently lead to right heart failure or even death if left untreated (McLaughlin et al., 2009). In the past few years, considerable clinical trials are commonly confined to controlling symptoms to prolong and improve the patient’s life, but PH is still incurable (Spaczynska et al., 2020). Hence, finding new effective drugs remains an urgent issue for the treatment of PH.

Nitric oxide (NO) has been revealed as critical messenger molecule in PH for long history and currently commonly used clinical drugs including Sildenafil, Tadalafil, and Riociguat exhibit therapeutical effects mainly by targeting on NO pathway. It is well known that NO binds to soluble guanylate cyclase (sGC) and increases the formation of cyclic guanosine monophosphate (cGMP) (Friebe et al., 2020). cGMP-dependent protein kinase I (cGKI) is one of the primary mediators of NO/cGMP-triggered signal transduction which is crucial in the regulation of vascular tension (Yang et al., 2013). During the past decades, experimental studies have demonstrated that sGC stimulator bear considerable potential benefits, including preventing or even reversing left ventricular hypertrophy and fibrosis, and reducing ventricular afterload through systemic and pulmonary vasodilation (Armstrong et al., 2018). For example, Riociguat, the first clinically available sGC stimulator, has been approved for PH and non-operable or recurrent/persistent chronic thromboembolic PH (Leuchte et al., 2015). HLQ2g is a new compound from our group, which is modified from the structure of Riociguat, with pyrazolpyridine ring (anti-fibrosis functional group) and pyrimidine ring (stimulating sGC functional group) in its structure. Similar to Riociguat, HLQ2g can regulate sGC/cGMP signaling pathway in PH model (Hu et al., 2020), but the specific mechanism of HLQ2g in the PH treatment remains to be elucidated.

BMPR2 is a serine/threonine membrane receptor. After being activated by bone morphogenetic protein (BMP) ligand, BMPR2 transfers from the cell membrane into the cytoplasm, and activates the phosphorylation of Smad protein in the cytoplasm (mainly Smad1/5/8). Subsequently, the complex formed by phosphorylated Smad1/5/8 (p-Smad1/5/8) with Smad protein, enters the nucleus, and initiates the transcription of downstream genes such as Id protein (Evans et al., 2016). Since 2000, numerous studies have revealed the critical role of BMP signaling pathway in PH. These studies found that BMPR2 gene mutations exist in 70% of patients with hereditary PH and 10–40% of patients with idiopathic PH (Atkinson et al., 2002; Yang et al., 2005). Down-regulation of the BMP signaling pathway is considered to be a key pathological mechanism affecting pulmonary vascular remodeling. Animal experiments have further found that the therapeutic effects of existing PH therapeutic drugs (such as sildenafil, prostacyclin, etc.) are related to the up-regulation of the BMP signaling pathway (Yang et al., 2013). Moreover, FK506 and BMP9 can reverse the development of PH by activating the BMP signaling pathway in animal models (Spiekerkoetter et al., 2013; Long et al., 2015). More importantly, it has been reported that crosstalk between cGKI and BMP signaling pathway are important mechanism hallmarks in PH (Schwappacher et al., 2009).

Therefore, we hypothesized that the novel pyrazolo[3,4-b] pyridine derivative (HLQ2g) could elevate cGMP level by increasing sGC activity, and thus activate cGKI and ultimately up-regulate the BMP signaling pathway. This study aims to reveal the mechanism of HLQ2g in the treatment of PH *in vivo* and *in vitro* and investigate the involvement of cGKI and BMP signaling pathway.

## Materials and Methods

### Animal Experiment

Hunan Normal University Experimental Animal Welfare Ethics Committee and Animal Management and Committee approved all animal experimental protocols. Male healthy Sprague Dawley rats (100–150 g) were from Hunan SJA experimental animal Co., Ltd. (No.: SYXK [Changsha] 2015-0017). All animals were reared in a controlled temperature (18°C–25°C) and humidity (50–60%), and 12-h light/dark cycle with free food and water. After 1 week of adaptive feeding, the rats were weighed, arbitrarily grouped, and numbered. Hypoxia-induced PH rat model was established and assigned into control, Hypoxia, Riociguat, and HLQ2g (CN 2019104923511) groups, with 10 rats in each group. The modeling time was 4 weeks. In the first week, rats in the Hypoxia, Riociguat, and HLQ2g groups were raised in a hypoxia box (oxygen concentration was set at 10%). From the third week, rats in the Riociguat and HLQ2g groups were given by gavage with the dosage of 10 mg/kg/d (Lang et al., 2012) (the solvent was sodium carboxymethyl cellulose, CMC-Na) for 2 weeks. There was no intervention in the control rats.

### Hemodynamic Measurements and Morphologic Analyses

After PH modeling, rats were anesthetized by 1% pentobarbital sodium (i.p., 50 mg/kg). A polyethylene catheter was inserted through the right jugular vein to the right atrium to record the RV systolic pressure (RVSP). After that, lung tissue samples were collected. The RV, left ventricle, and interventricular septum (LV + S) were separated and weighed to calculate the mass ratio of RV/(LV + S). The tibia of the hind limbs of rats was removed and the distance was measured with a ruler to calculate the RV/tibial length. The right lower lung was removed and fixed in 4% paraformaldehyde solution, and then for subsequent vascular morphological analysis. The tissue sections were kept at 60°C for 2 h and then in xylene solution for 30 min. Then the sections were put into ethanol solution (100, 95, 70%) successively for 5 min each time. Next, the samples were washed in phosphate-buffered saline (PBS, Procell, China), for 3 times, 5 min each time. Next, the sections were kept for 5 min in high pressure antigen repair and 20 min in hydrogen peroxide. Hematoxylin-Eosin (HE) staining was used to observe the morphology of blood vessels. Structure remodeling of pulmonary arterioles was observed under light microscope (Nikon, Japan). Pulmonary arterioles with 50–150 µm diameter were arbitrarily observed and analyzed by Image-Pro Plus 6.0. The ratio of media wall thickness (WT%) = (outside diameter-inside diameter)/(outside diameter) × 100.

### Cell Culture

Human pulmonary artery smooth muscle cells (HPASMCs, ScienCell, United States) were cultured in high glucose DMEM (Gibco, United States) containing 20% fetal bovine serum (FBS, Biological Industries, Israel) and 1% penicillin/streptomycin (HyClone, United States) in an incubator (5% CO_2_, 37°C). Cells at passage 3 to 10 were used for the experiment.

### HTRF cGMP Assays

The cGMP accumulation assays in HPASMCs followed standard protocols. Homogeneous time-resolved fluorescence (HTRF) cGMP assay was performed according to the cGMP kits (Cisbio). HPASMCs were resuspended in PBS containing 1 mM PDE-5 inhibitor 3-isobutyl-1-methylxanthine (IBMX) and 0.2% BSA at 1 × 10^5^ cells/ml with or without 10 μM sGC inhibitor 1H-[1,2,4]oxadiazolo [4,3-a] quinoxalin-1-one (ODQ), and allocated into 384-well plates (HTRF^®^) at 5 μl/well. Test compounds were solubilized to 100 mM in DMSO and serially diluted by the diluent of the cGMP kits to achieve a 2 × stock, which was diluted using 10-fold dilutions to produce a 6-point dose-response curve with a top concentration of 200 μM. Diluted compounds were then transferred to a triplicate set of assay plates (5 μl/well). After 1 hour incubation, 5 μl cGMP-d2 reagent diluted in lysis buffer was added to each well followed by 5 μl europium cryptate reagent. Next, the plates were mounted and incubated for 1 hour before reading on an HTRF^®^ compatible reader (Bio Tek, United States).

### Cell Counting Kit-8 Assay

HPASMCs were plated into 96-well plates until the final concentration was 3,000 cells/well. After cell adhesion, HPASMCs were cultured in serum-free medium containing stimulating factors, and allocated into control, platelet-derived growth factor (PDGF) (20 ng/ml), PDGF + Riociguat (1 μM), PDGF + HLQ2g (0.5 μM), PDGF + HLQ2g (1 μM), Hypoxia, Hypoxia + Riociguat (1 μM), Hypoxia + HLQ2g (0.5 μM), Hypoxia + HLQ2g (1 μM) and Hypoxia + HLQ2g (1 μM) + Rp-8-Br-PET-cGMPS (30 μM) groups. HPASMCs in the Hypoxia, Hypoxia + Riociguat (1 μM), Hypoxia + HLQ2g (0.5 μM), Hypoxia + HLQ2g (1 μM) and Hypoxia + HLQ2g (1 μM) + Rp-8-Br-PET-cGMPS (30 μM) groups cultured in hypoxia incubator (3% O_2_, 5% CO_2_, 37°C) for 24 h, while other groups of cells were cultured in normal incubators for 24 h. Then culture medium was replaced with fresh medium containing 10 μl CCK8 (Dojindo, Japan). After incubation at 37°C for 2 h, the optical density at 450 nm was read using a microplate reader (ThermoFisher, United States). Six replicates were used for each treatment.

### 5-Ethynyl-2′-Deoxyuridine Assay

HPASMCs were plated into 96-well plates until the final concentration was 5,000 cells/well. After cell adhesion, HPASMCs were cultured in serum-free medium containing stimulating factors, and allocated into control, platelet-derived growth factor (PDGF) (20 ng/ml), PDGF + Riociguat (1 μM), PDGF + HLQ2g (1 μM), Hypoxia, Hypoxia + Riociguat (1 μM) and Hypoxia + HLQ2g (1 μM) groups. HPASMCs in the Hypoxia, Hypoxia + Riociguat (1 μM) and Hypoxia + HLQ2g (1 μM) groups cultured in hypoxia incubator (3% O_2_, 5% CO_2_, 37°C) for 24 h, while other groups of cells were cultured in normal incubators for 24 h. Then culture medium was replaced with fresh medium containing 10 μM EdU (Beyotime, China). After incubation at 37°C for 3 h, the staining treatment was carried out. Three replicates were used for each treatment.

### Scratch Test

Cell scratch test was used to detect HPASMCs migration. In short, a 6-well plate was labeled on the back with evenly distributed horizontal lines at 1 cm interval. Then 5 × 10^5^ cells were plated in each well, and starved overnight until reaching 70–80% confluence. After that, three parallel lines were randomly scratched using a 1 ml pipette. HPASMCs were washed with PBS and cultured in serum-free medium (DMEM, Gibco, United States) containing Riociguat (1 μM) or HLQ2g (1 μM). Then, HPASMCs were cultured in hypoxia incubators (3% O_2_, 5% CO_2_, 37°C), and photographed at 0 and 24 h after culture. The densitometric quantification was conducted with Image J 1.43 (NIH, United States).

### Transwell Assay

HPASMCs were seeded into Transwell chamber. The volume of serum-free medium was 200 μl, and the number of cells in each well was 5 × 10^4^. Cells were assigned into control, Hypoxia, Hypoxia + HLQ2g (1 μM), and Hypoxia + HLQ2g (1 μM) + Rp-8-Br-PET-cGMPS (30 μM) groups. The cells were cultured in the incubator for 24 h and then wiped off with cotton swabs. Then the cells were washed with PBS three times, fixed 30 min with 4% paraformaldehyde, and stained for 15 min with 0.4% crystal violet. Finally, the crystal violet dye was removed. The number of cells passing through the polycarbonate membrane was observed and calculated under Nikon microscope.

### Western Blot Analysis

The proteins were isolated from pulmonary arteries or HPASMCs using radio-immunoprecipitation assay buffer (containing 0.1% phenylmethylsulfonyl fluoride), and equal proteins (30 μg) were separated by electrophoresis and moved to polyvinylidene fluoride membranes. Then the membranes were blocked with 1% BSA for 1 h, followed by overnight incubation with primary antibodies against cGKI (CST, United States), BMPR2 (Proteintech, United States), p-Smad1/5/8 (CST, United States), Id1 (Proteintech, United States), PCNA (CST, United States), and β-actin (Proteintech, United States) at 4°C. After that, the membranes were probed with HRP-labeled secondary antibodies (Jackson, United States). The signals of bands were measured using Luminata Creseendo Western HRP Substrate (Millipore) through Molecular Imager ChemiDoc XRS System (Bio-Rad, United States). The protein levels were quantified using Image J 1.43.

### Statistical Analysis

Statistical analysis was conducted by SPSS 18.0 (IBM Corp, United States). The results were described as mean ± standard deviation (SD) and analyzed using one-way analysis of variance (ANOVA) followed by Newman-Keuls test for multiple comparisons. *p* < 0.05 was considered significant.

## Results

### HLQ2g Up-Regulated Intracellular cGMP Level by Stimulating sGC

**FIGURE 1:**
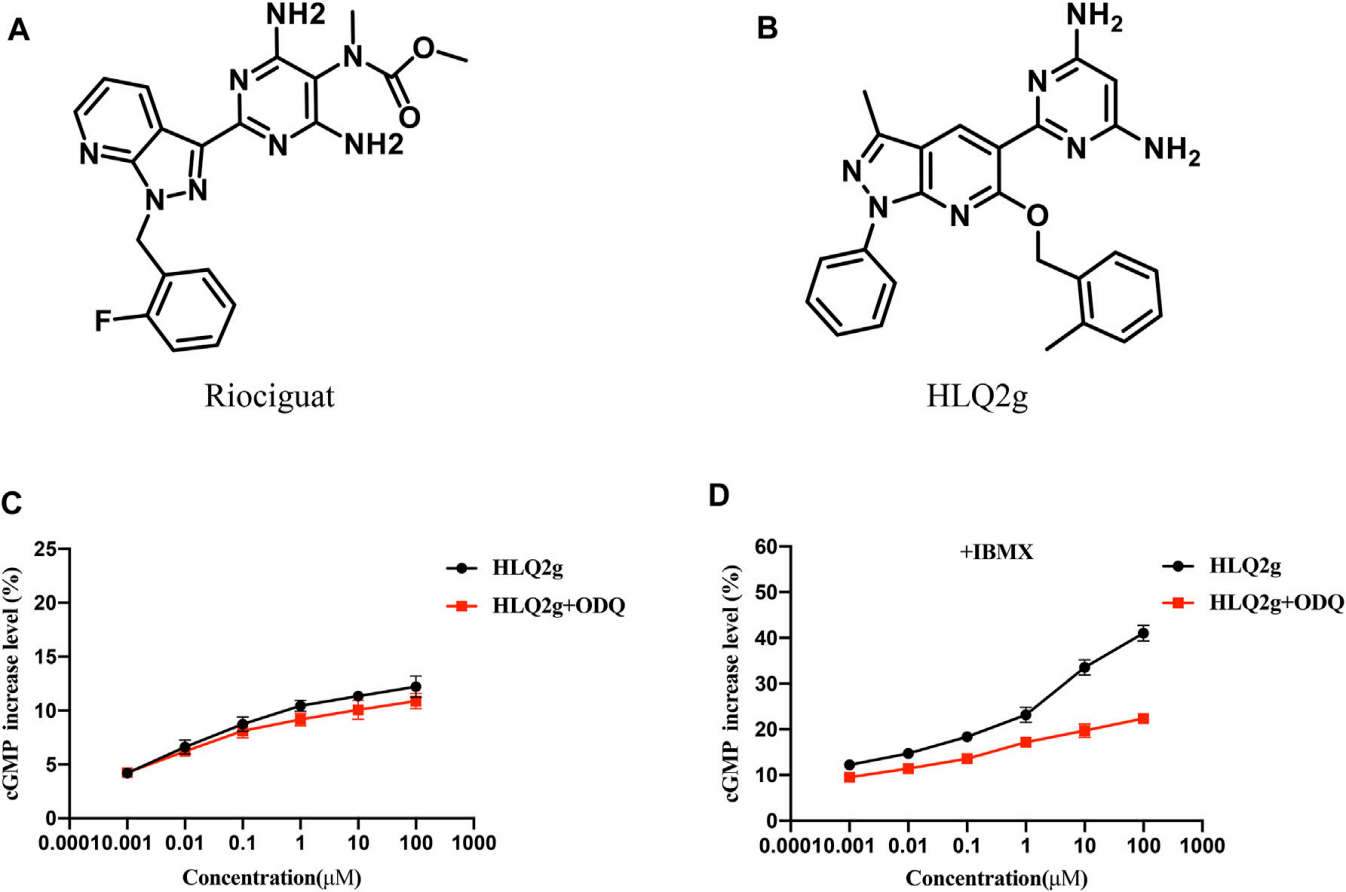
HLQ2g can stimulate sGC and upregulate cGMP. **(A)**: The structure of Riociguat. **(B)**: The structure of HLQ2g. **(C)**: Dose-dependent curve of HLQ2g to elevate the cGMP levels in the absence or presence of 10 μM ODQ in HPASMCs without IBMX, values are the average of three independent experiments. **(D)**: Dose-dependent curve of HLQ2g to elevate the cGMP levels in the absence or presence of 10 μM ODQ in HPASMCs under condition of IBMX (10 μM), values are the average of three independent experiments.

sGC signal transduction is crucial for vascular tone modulation in PH pathogenesis. Pharmacological stimulation of sGC have intracellular effects by augmenting the formation of cGMP that can be degraded by phosphodiesterase 5 (PDE-5). To verify whether HLQ2g can activate sGC, we use HTRF cGMP assay to evaluate the effect of HLQ2g on cGMP generation in HPASMCs. Firstly, in the absence of IBMX (PDE-5 inhibitor), HLQ2g slightly enhanced intracellular cGMP levels (about 5% increase at 100 μM, Figure 1C). The presence or absence of ODQ (sGC inhibitor) had no significant difference on the levels of cGMP (Figure 1C). This is mainly due to the rapid cGMP degradation catalyzed by the intracellular PDE-5. Secondly, with the addition of IBMX, we investigated the influence of ODQ on cGMP formation in HPASMCs. Consequently, HLQ2g showed distinct ability to produce cGMP in a significant dose-dependent manner (Figure 1D). In addition, the elevated levels of cGMP were clearly reduced after adding ODQ compared with the groups without ODQ (Figure 1D). Taken together, these findings suggest that HLQ2g was effective in elevating the intracellular cGMP levels possibly through stimulating sGC.

### HLQ2g Reduced RVSP and RV Hypertrophy and Improved Vascular Remodeling in Hypoxia-Induced PH Rat Model

**FIGURE 2:**
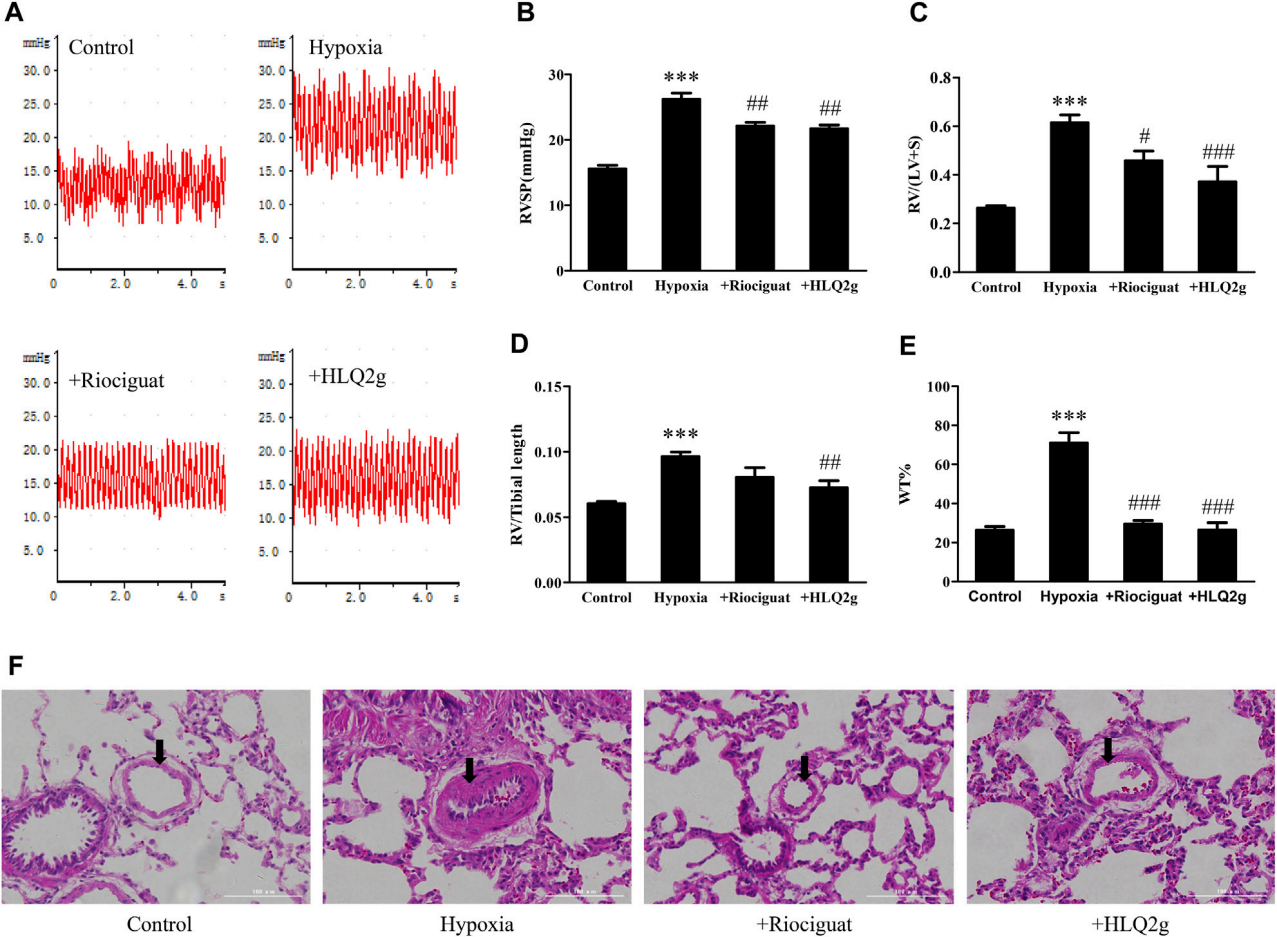
Hemodynamics, right heart remodeling, pulmonary vascular remodeling in Hypoxia-induced PH rats. **(A)**: RVSP waveform; **(B)**: RVSP summary graph; **(C)**: RV/LV + S (**right** ventricle/**left** ventricle + interventricular septum) statistics; **(D)**: RV/Tibial length (**right** ventricle/tibial length) statistics; **(E)**: Statistical chart of HE staining (100 μm); (WT%) = (outside diameter-inside diameter)/(outside diameter) × 100. **(F)**: Representative image of HE staining. Control: the control rats; Hypoxia: Hypoxia-induced PH rats. Data are expressed as mean ± SD. *n* = 3–5. Vs. the control, ***p < 0.001; vs. the Hypoxia group, #*p* < 0.05, ##*p* < 0.01, ###*p* < 0.001.

To explore the role of HLQ2g *in vivo*, we established a PH rat model induced by hypoxia. Hypoxia significantly increased RVSP, and the administration of Riociguat and HLQ2g notably inhibited the increase of hypoxia-induced RVSP (Figures 2A,B). The RV hypertrophy index (RVHI) in the hypoxia group was higher than that in the normal group; and the RVHI in the Riociguat group and HLQ2g group was lower than that in the hypoxia group (Figures 2C,D). HE staining indicated that compared with the normal group, the vascular remodeling index (WT%) in the hypoxia group was clearly increased; compared with the hypoxia group, the vascular remodeling index of Riociguat group and HLQ2g group was obviously decreased (Figures 2E,F). These results indicate that hypoxia can induce PH successfully, and HLQ2g can reduce RVSP and cardiac hypertrophy and improve vascular remodeling.

### HLQ2g Up-Regulated cGKI Expression and Restored BMPR2 Signaling Pathway in Hypoxia-Induced PH Rat Model

**FIGURE 3:**
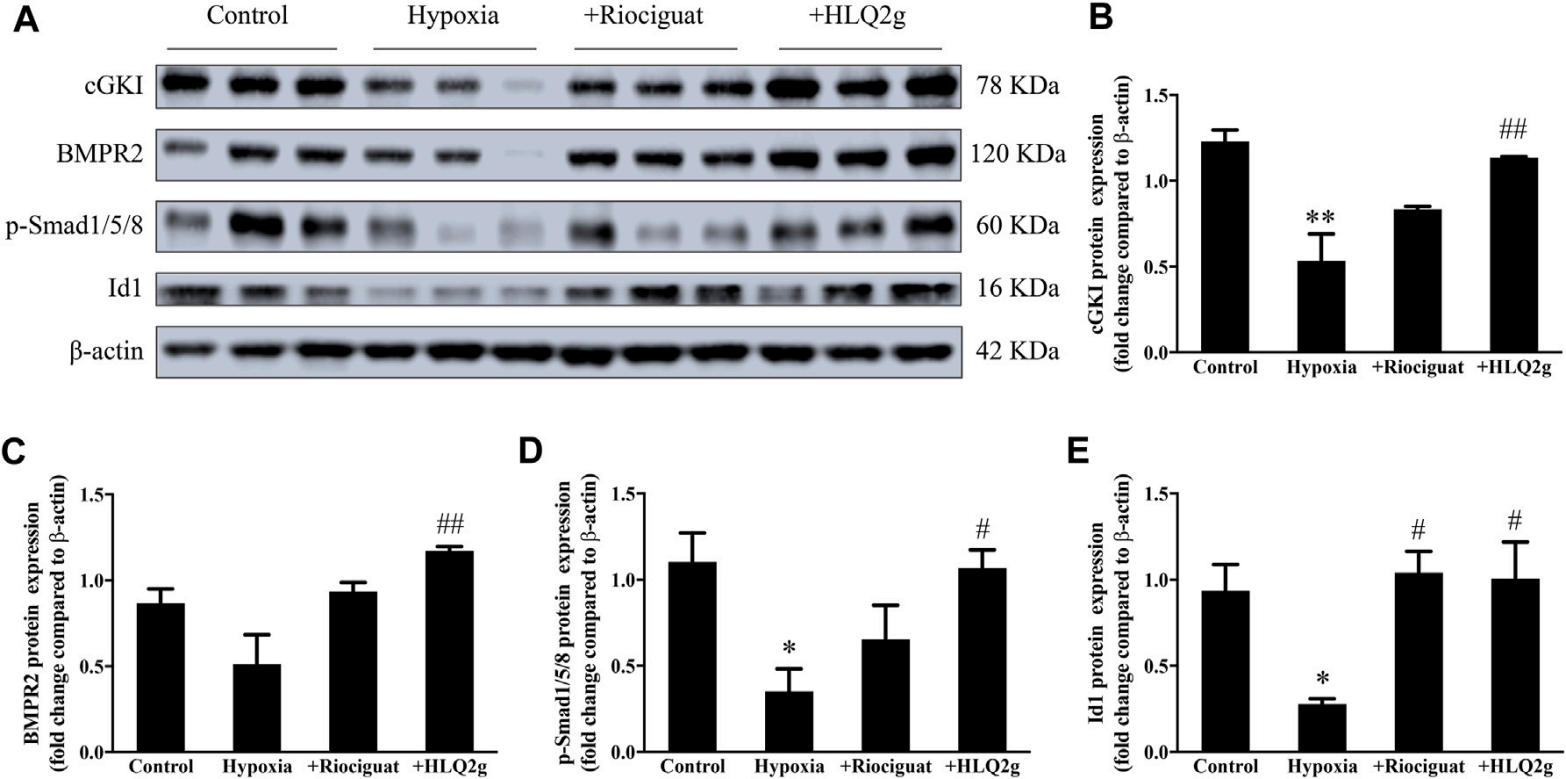
In hypoxia-induced PH rat model, HLQ2g can regulate cGKI protein and BMPR2 pathway. **(A)**: The expression of cGKI, BMPR2, p-Smad1/5/8, and Id1 in pulmonary artery of rats; **(B–E)**: The quantitative analysis results of A. Control: the control rats; Hypoxia: Hypoxia-induced PH rats. Data are expressed as mean ± SD. *n* = 3. Vs. the control, *p < 0.05, **p < 0.01; vs. the Hypoxia group, #*p* < 0.05, ##*p* < 0.01.

To further explore the role of HLQ2g in the regulation of mechanism *in vivo*, we detected cGKI expression and BMPR2 signaling pathway in pulmonary artery of hypoxia-induced PH rat model. The down-regulation of cGKI, p-Smad1/5/8, and Id1 protein levels induced by hypoxia was significantly restored after HLQ2g administration (Figures 3A–E). These results suggest that HLQ2g could upregulate cGKI protein and BMPR2 signaling pathway in hypoxia-induced PH rat model.

### HLQ2g Inhibited Proliferation and Migration of HPASMCs

**FIGURE 4:**
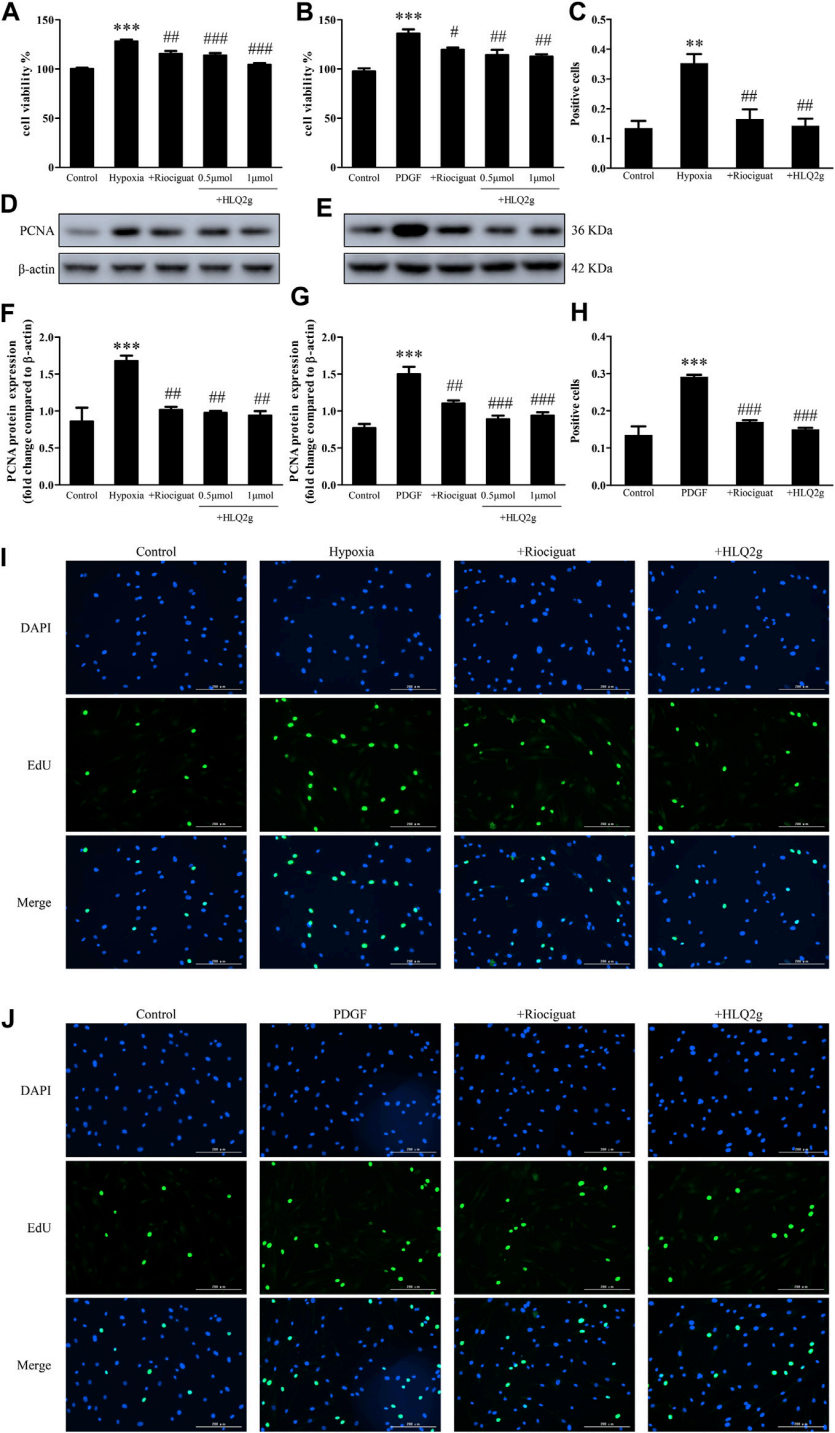
HLQ2g can inhibit the proliferation of HPASMCs. **(A, B)**: CCK8 detected cell proliferation. **(C)**: Quantitative analysis of I. **(D)**: The expression of PCNA protein in HPASMCs stimulated by hypoxia. **(E)**: The expression of PCNA protein in HPASMCs induced by PDGF. **(F)**: Quantitative analysis of D. **(G)**: Quantitative analysis of E. **H**: Quantitative analysis of J. **(I, J)**: EdU detected cell proliferation. Riociguat (1 μM); HLQ2g (1 μM); **Vehicle**: 1% DMSO; **Control**: normal control group; **PDGF**: platelet-derived growth factor BB treatment group (20 ng/ml; 24 h). Data are expressed as mean ± SD, *n* = 3–5. Vs. the control, **p < 0.01, ***p < 0.001; vs. the PDGF/Hypoxia group, #*p* < 0.05, ##*p* < 0.01, ###*p* < 0.001.

Excessive proliferation and migration of HPASMCs play an important role in the vascular remodeling of PH. To detect the effect of HLQ2g on the proliferation of HPASMCs, HPASMCs were induced with hypoxia and incubated with HLQ2g for 24 h, and then the cell proliferation was measured. CCK8 and EdU results showed that hypoxia induced the proliferation of HPASMCs, while HLQ2g inhibited the proliferation of HPASMCs induced by hypoxia (Figures 4A,C,I). Similarly, HLQ2g inhibited the proliferation of HPASMCs induced by PDGF (Figures 4B,H,J). We also examined the effect of HLQ2g on the proliferation of HPASMCs at protein level. The results revealed that PCNA protein was upregulated after hypoxia stimulation and downregulated after HLQ2g intervention (Figures 4D,F). We also used PDGF to stimulate HPASMCs, and the results were consistent with hypoxia (Figures 4E,G). These results suggest that HLQ2g could inhibit the proliferation of HPASMCs.

**FIGURE 5:**
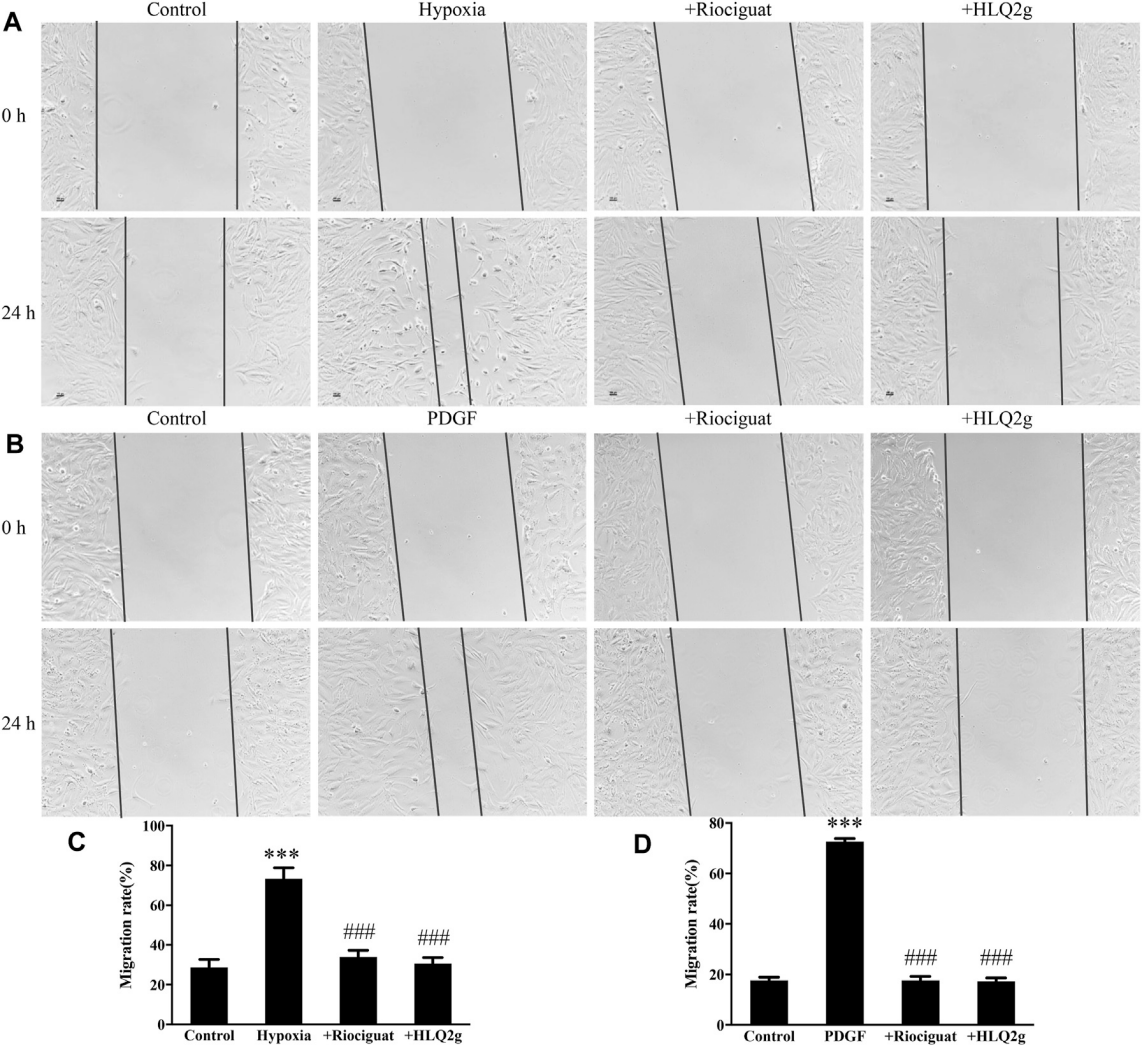
HLQ2g can inhibit the migration of HPASMCs. **(A, B)**: Scratch test results (100 μm); **(C)**: Statistical analysis of A; **(D)**: Statistical analysis of B. Riociguat (1 μM); HLQ2g (1 μM). Data are expressed as mean ± SD, *n* = 3. Vs. the control, ***p < 0.001; vs. the PDGF/Hypoxia group, ###*p* < 0.001.

At the same time, we conducted scratch tests to detect the effect of HLQ2g on HPASMCs migration. Hypoxia induced PASMC migration, while HLQ2g inhibited the migration of PASMC induced by hypoxia (Figures 5A,C). The migration of HPASMCs induced by PDGFs was inhibited by HLQ2g (Figures 5B,D). These results suggest that HLQ2g could inhibit the proliferation and migration of HPASMCs induced by hypoxia and PDGF.

### HLQ2g Up-Regulated cGKI Expression and Restored BMPR2 Signaling Pathway in HPASMCs

**FIGURE 6:**
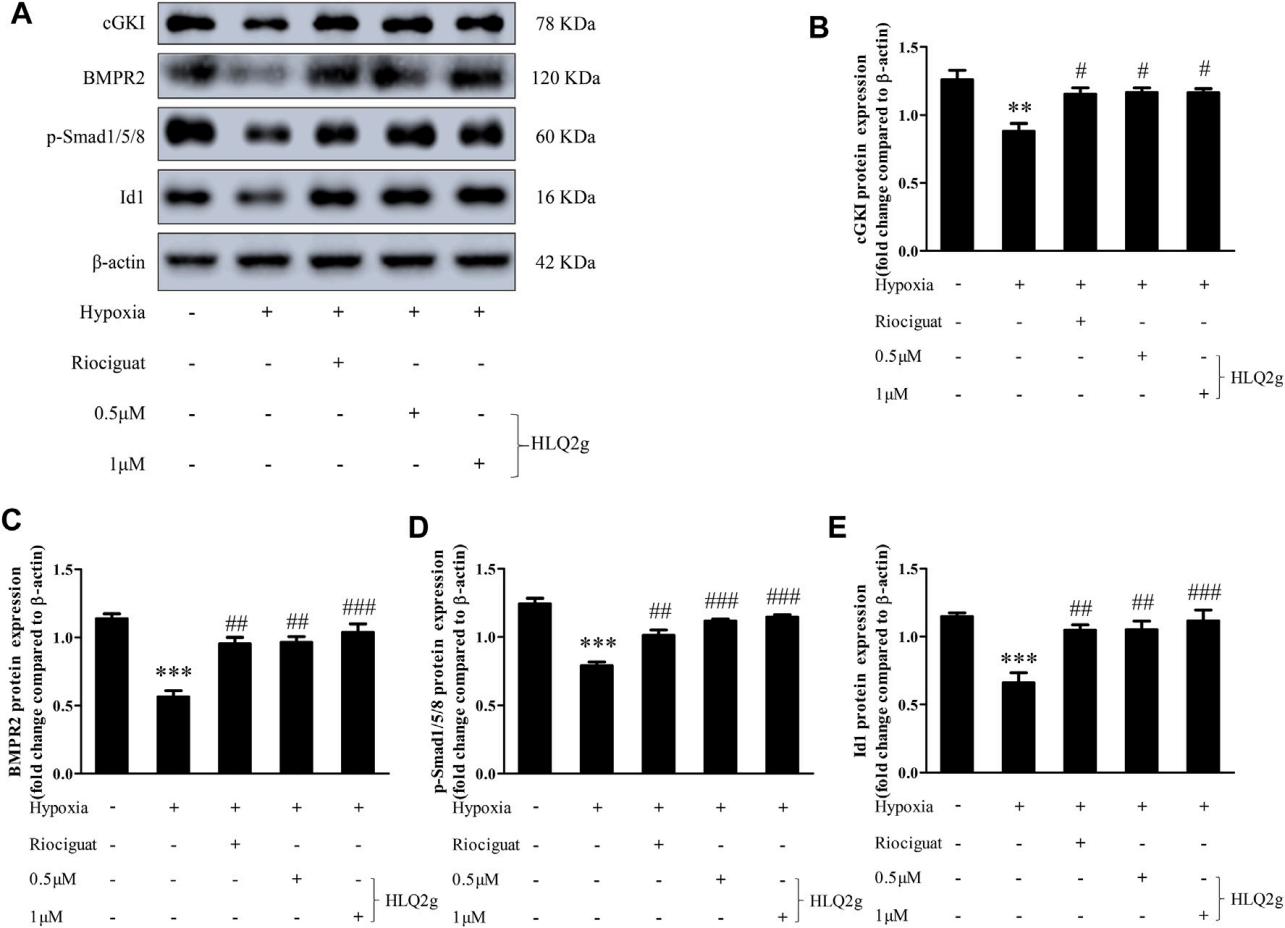
HLQ2g can upregulate cGKI expression and BMP signaling pathway in Hypoxia-induced HPASMCs. **(A)**: The protein expression of cGKI, BMPR2, p-Smad1/5/8, and Id1 in HPASMCs determined by Western blot. Riociguat (1 μM). **(B–E)**: Quantitative analysis results of graph A. Data expressed as mean ± SD, *n* = 3. Vs. the control, **p < 0.01, ***p < 0.001; vs. the Hypoxia group, #*p* < 0.05, ##*p* < 0.01, ###*p* < 0.001.

To further explore the role of HLQ2g in the regulation of mechanism *in vitro*, HPASMCs were induced with hypoxia and incubated with HLQ2g for 24 h, and then the intracellular protein levels were detected. Hypoxia significantly downregulated cGKI protein and the BMPR2 pathway, while HLQ2g intervention significantly inhibited the downregulation of cGKI, BMPR2, p-Smad1/5/8, and Id1 induced by hypoxia (Figures 6A–E). In short, HLQ2g could upregulate cGKI and BMPR2 pathway in HPASMCs.

### cGKI Inhibition Abrogated the Effects of HLQ2g on BMP Signaling Pathway in HPASMCs

**FIGURE 7:**
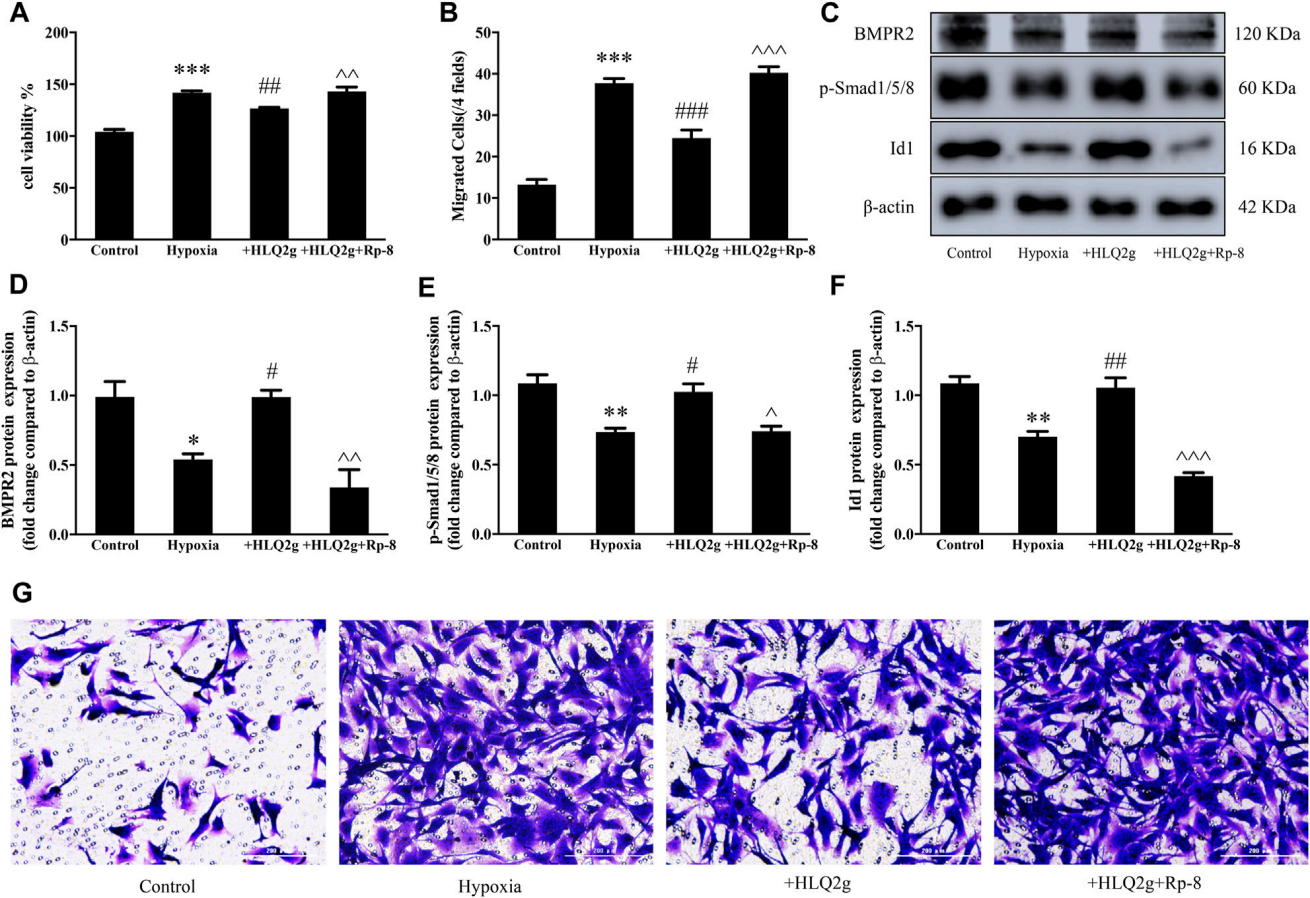
Rp-8-Br-PET-cGMPS can block the effect of HLQ2g. **(A)**: CCK8 detected cell proliferation. **(B, G)**: Transwell test results (200 μm). **(C)**: The protein expression of BMPR2, p-Smad1/5/8, and Id1 in HPASMCs determined by Western blot. **(D–F)**: Quantitative analysis results of graph C. HLQ2g (1 μM); Rp-8: Rp-8-Br-PET-cGMPS (30 μM). Data expressed as mean ± SD, *n* = 3–5. Vs. the control, *p < 0.05, **p < 0.01, ***p < 0.001; vs. the Hypoxia group, #*p* < 0.05, ##*p* < 0.01, ###*p* < 0.001; vs. the Hypoxia + HLQ2g group, ^*p* < 0.05, ^^*p* < 0.01, ^^^*p* < 0.001.

To confirm our hypothesis, HPASMCs were induced with hypoxia and incubated with both HLQ2g and Rp-8-Br-PET-cGMPS (a specific cGKI inhibitor), then cell proliferation and migration were detected, and the intracellular protein levels were detected. The results showed that adding Rp-8-Br-PET-cGMPS promoted cell proliferation and migration and down-regulated the BMPR2 pathway, compared with using HLQ2g alone (Figures 7A–G). These results suggest that Rp-8-Br-PET-cGMPS blocked the effects of HLQ2g, which was proved to up-regulate BMP signaling pathway by activating cGKI, thus inhibiting cell proliferation and migration.

## Discussion

In summary, the present study found that HLQ2g can reduce RVSP and inhibit the development of RV hypertrophy and pathological changes of pulmonary vascular remodeling in hypoxia-induced PH rat model. At the same time, HLQ2g restored the BMP pathway by up-regulating cGKI in PH. In addition, HLQ2g inhibited hypoxia and platelet-derived growth factor-induced proliferation and migration of HPASMCs which was abolished by cGKI inhibitor. In short, this study preliminarily revealed the therapeutic mechanism of the novel pyrazolo[3,4-b] pyridine derivative (HLQ2g), and also further proved the interaction between NO signaling and BMP signaling which gave us important clues in PH therapy.

PH remains a serious clinical disease regardless of the availability to a variety of drugs interfering with endothelin, NO, and prostacyclin pathways in the past decades (Galie et al., 2019). NO/sGC/cGMP was accepted as a classic target pathway for the treatment of PH by modulating a variety of downstream targets including protein kinase, cyclic nucleotide-gated channel, and phosphodiesterase (Lundberg et al., 2015). Current medicines including sildenafil, tadalafil, and riociguat are commonly used in clinical mainly by increasing the cGMP level and causing pulmonary arteries relaxation. Different from other drugs which inhibit the degradation of cGMP, riociguat is the first approved sGC stimulator in 2013 to accelerate the production of cGMP. Since that time, exploring sGC stimulators and sGC activators have became hot spot in this fields, and some of them have been under clinical trials (Sandner et al., 2019). HLQ2g is a new compound modified from the structure of Riociguat by our group, which preserved the active fragment (pyrimidine and benzyl moieties) of riociguat to ensure its activity of activating sGC, which could achieve the vasodilation effect. In addition, the active fragment (pyrazolo[1,5-a] pyrimidine) of AMPK inhibitor was introduced in HLQ2g to increase the anti-proliferative activity. As a result, HLQ2g showed potential advantages with dual regulatory activities on vascular remodeling and vasodilation compared with riociguat. Previous study has revealed the comparable pharmacological effects of HLQ2g and Riociguat in PH treatment (Hu et al., 2020). This study further confirmed the cGMP elevation and therapeutical functions of HLQ2g *in vivo* and *in vitro* which defined the critical role of sGC/cGMP pathway in PH treatment again.

Firstly, we established a rat model of PH induced by hypoxia. It is well known that vasocontraction and vascular remodeling are considered as the primary pathological characteristic of PH (Humbert et al., 2004). Chronic hypoxia-induced right ventricular hypertrophy is mainly caused by mechanical stress of ventricular wall resulted from PH (Tual et al., 2006). Our data exhibited that RVSP, RVHI, and vascular remodeling index were significantly down-regulated in hypoxia-induced PH rats after the administration of HLQ2g. These solid proofs identified the therapeutic effects of HLQ2g in PH rat models. Furthermore, we conducted cell culture to explore the effects of HLQ2g *in vitro*. It is well accepted that the excessive proliferation and aberrant migration of PASMCs are critical abnormal phenotypes of PH, which largely contributes to the thickened pulmonary vascular wall and eventually lead to vascular occlusion (Li et al., 2016; Hu et al., 2020). HPASMCs were treated with hypoxia and incubated with HLQ2g for 24 h, and cellular proliferation and migration were investigated. These results revealed that HLQ2g inhibited the proliferation and migration of HPASMCs induced by hypoxia. Besides, PDGF was also used to induce proliferation and migration of HPASMCs as it is acknowledged as a mitogen to PASMCs and further facilitate pulmonary vascular remodeling (Li et al., 2018). Similarly, we found that HLQ2g inhibited the proliferation and migration of HPASMCs stimulated by PDGF. Taken together, these results indicate that HLQ2g can inhibit vascular remodeling and exert a considerable therapeutic effect on PH.

To explore the specific mechanism of HLQ2g, we turned our sight to the BMP signaling pathway. It is reported that mutations in BMPR2 result in the deficiency of the growth-inhibitory effects of BMPs in HPASMCs through a downregulated Smad1/5/8 phosphorylation and reduced transcription of Inhibitor of DNA binding protein 1, which is a primary target gene of BMP signaling (Machado et al., 2006). Dysfunction of BMP signaling is thought to be an important hallmark in various types of PH (Long et al., 2009). Notably, animal experiments have further found that the therapeutic effects of existing PH therapeutic drugs (such as sildenafil, prostacyclin, etc.) are related to the up-regulation of the BMP signaling pathway (Yang et al., 2013). Direct activation of BMPR2 and BMP signaling by BMP9 or FK506 have showed powerful effects on PH treatment in animal models and clinical transformation research is ongoing. Thus, the BMP signaling pathway has enormous potential to become next therapeutical target in PH. Here, this study showed that down-regulated protein expressions of BMPR2, p-Smad1/5/8, and Id1 protein were observed in rats and HPASMCs treated by hypoxia, while HLQ2g treatment notably restored the BMP signaling pathway. Up-regulated BMP signaling pathway is assumed to participate into the improvements of vascular remodeling and PH. Meanwhile these novel findings attracted us to investigate the relationship between sGC activation and BMP signaling pathway.

As a key mediator of vasodilation, cGKI is mainly activated by cGMP. Interestingly, there is research reported that cGKI can regulate BMP receptor and Smads and enhance BMP signal transduction (Schwappacher et al., 2009). As a sGC stimulator and cGMP elevator, there is reason to image the role of cGKI in HLQ2g’s biological function. Results from this study demonstrated that HLQ2g treatment notably renewed hypoxia-induced down-regulation of cGKI in rats and HPASMCs, confirming the up-regulation effects of HLQ2g on cGKI probably via elevated cGMP. Interestingly, when we interfered cGKI with Rp-8-Br-PET-cGMPS in HPASMCs, we found that the ability of HLQ2g to inhibit the proliferation and migration of HPASMCs and upregulate the BMP signaling pathway was significantly suppressed. It means that Rp-8-Br-PET-cGMPS blocks the effects of HLQ2g, which has been proved to restore BMP signaling pathway and inhibit vascular remodeling. The experimental results proved the direct interaction between sGC signaling and BMP signaling, and suggested that BMP/Smad/Id1 pathway is the key effector in hypoxic PH.

## Conclusion

In conclusion, the results in this study demonstrated that HLQ2g inhibited the proliferation and migration of HPASMCs and alleviated the progression of PH by upregulating cGKI protein to activate the BMP signaling pathway. This study might shed lights on finding novel agents targeting on sGC and BMP signaling. Further study using *BMPR2* and *cGKI* knockout animal would be helpful to further prove the relationship between sGC signaling and BMPR2 signaling in PH. Furthermore, previous study suggested an anti-fibrosis effects of HLQ2g in PH which may partly attribute to inhibition of AMPK pathway. Actually, the unbalance of BMP and TGF-beta also play a key role in vascular fibrosis which worth further investigation. Importantly, we shall conduct more researches on the feasibility and safety of HLQ2g in the treatment of PH, and hope to push it into clinical trials.

## Data Availability

The original contributions presented in the study are included in the article/Supplementary Material, further inquiries can be directed to the corresponding author.
